# Food insecurity among consumers from rural areas in Romania

**DOI:** 10.3389/fnut.2023.1345729

**Published:** 2024-01-15

**Authors:** Carmen Adina Paştiu, Silvia Ştefania Maican, Iulian Bogdan Dobra, Andreea Cipriana Muntean, Camelia Haţegan

**Affiliations:** ^1^Faculty of Economics Science, 1 Decembrie 1918 University of Alba Iulia, Alba Iulia, Romania; ^2^Faculty of Economics Science, West University of Timişoara, Timişoara, Romania

**Keywords:** food insecurity, consumers, food consumption, food availability, food access

## Abstract

Food security has a special relevance in nowadays economies, due to the current crisis, characterized by multiple layers on a social, political, economic, and individual biological level. The present study aims to identify relevant aspects of food insecurity for consumers in rural Romania and the main factors that significantly influence it (food availability, food access, and food consumption). The data were collected from a sample of 875 consumers from rural areas in Romania. The results show that food insecurity is perceived by the consumers of Romanian rural households as being strongly influenced by food availability, but less influenced by food consumption and access. The results have an essential relevance in the development of agri-food marketing strategies and public policies in the field of sustainable development.

## 1 Introduction

Food security is a global concern given global population growth, climate change that can affect agricultural production, globalization, international trade and dependence on food imports for more vulnerable countries, difficult access to food due to economic and social inequalities, the international political environment and the global trend toward changes in food preferences and lifestyle.

According to the Food and Agriculture Organization (1, 2), these factors influencing food security can be grouped into four categories: availability—related to food supply, and access to available food products, consumption and stability—the constant availability, access, and use of food resources over time.

Shaw (3) pointed out that: “Food security exists when all people, at all times, have physical and economic access to sufficient, safe and nutritious food that meets their dietary needs and food preferences for an active and healthy life”.

For rural consumers in Romania, this trend of globalization and the development of the global economy have led to an increased homogeneity of diets, with food products being disconnected from their source. This has resulted in increased uncertainty in the supply chain, making it susceptible to disruptions and causing food insecurity (4, 5).

In agri-food marketing, the identification of vulnerable groups of consumers is essential to satisfy their needs, regardless of the country's level of development. First of all, agricultural products are intended to satisfy a physiological need (survival), according to Maslow's pyramid of needs (6). The most basic need is for physical survival, and this will be the first thing that motivates consumer behavior.

In Romania, according to data provided by the National Institute of Statistics in 2018 (7), approximately 47% of the country's population lives in rural areas, and a significant part of them produce their food in their households. The access of this category of consumers to food can be limited by various economic and social barriers.

Food insecurity differs among consumers in rural and urban areas in Romania. The existing research shows that there are important differences in food diversity between actual consumption and purchased food quantities, but these differences are not influenced by the residence area (urban vs. rural) (8). However, the analysis of food consumption patterns reveals that there is a higher consumption of main food products in urban areas compared to rural areas (9). Additionally, the study highlights that the rural population has higher expenditure elasticities for food demand compared to the urban population, mainly due to lower cash incomes (10). These findings suggest that food insecurity may be more prevalent in rural areas due to lower food diversity and lower cash incomes, which can affect access to food and threaten food security.

Food insecurity among consumers in rural areas of Romania is an important research topic. Analyzing how food consumption, food availability and food access relate to food insecurity among consumers from rural areas in Romania can help identify areas for improvement in food quality and living standards.

## 2 Materials and methods

### 2.1 Literature review. Theoretical framework and hypothesis development

Regarding food consumption, there are significant differences between rural and urban consumers. Food consumption patterns in rural and urban areas can differ significantly due to various factors, including lifestyle, access to resources, economic conditions, agricultural practices and cultural influences. The latter produce their food, while urban consumers buy it. Also, urban consumers have access to a greater diversity of food. Food consumption among rural consumers can be explained through several factors: price, as a key driver for purchasing behavior, social context and habits of food purchase and consumption, health concerns, awareness of the environmental impact, trends in food such as currents of vegetarianism and veganism, consumption of local products agricultural practices. A complex interplay of various factors influences the determinants of food and non-food consumption. According to Savadogo and Brandt (11), income, education, household size and structure are important determinants of food and non-food consumption.

Furthermore, studies have shown the relevance and the relations between food access and consumption as well as the important relationships between neighborhood food environment and consumption measures (12, 13). Consequently, based on the above opinions, the following hypothesis has been established:

H1: *There is a strong correlation between food consumption and food access*.

Food consumption in rural areas is closely tied to food security, as these areas often rely on local agricultural practices and the availability of homegrown or regionally produced food.

In his research, Skeratt (14), demonstrates that “place” has a significant impact on food consumption, as it influences the type of food available that affects consumer choices, and this can be observed by comparing consumers who live in the environment rural and those who live in the urban environment.

In 1986, Swaminathan presented the idea of “Nutrition Security”, which was defined as “physical, economic, and social access to a balanced diet, clean drinking water, environmental hygiene, primary health care, and nutritional literacy,” which has been emphasized. The term has three dimensions: availability, access, and absorption (15).

Availability describes the actual availability of food supplies in the appropriate amounts. Using food grains as a stand-in for food (fair enough in a situation where food grains make up a significant portion of caloric intake), the availability of food grains is determined by net domestic production plus net imports plus stock drawdown, net of feed, seed, and waste. Market integration within the borders of a country and storage and transportation infrastructure are prerequisites for physical availability in any given area.

The bundle of entitlements that relate to people's starting points, what they can obtain (particularly in terms of physical and financial access to food), and the opportunities that are available to them to attain entitlement sets with sufficient food—either through their efforts, through state intervention, or both—all determine *access*.

The capacity to use the food ingested for biological purposes is known as absorption. This in turn is closely tied to the availability of clean water for drinking, sanitation, a sanitary environment, primary healthcare, as well as suitable eating habits and information (16).

Even though during harvest time there usually are no problems with the availability of food, all around the globe, in each country some institutions work on ensuring food availability for their citizens. It focuses on the availability of enough food in acceptable quantities, whether it comes from imports or home production (17).

Additionally, scholars have outlined the status and the relations between food access and food availability among different types of consumers, as well as the influence of other factors (e.g., cost disparity, food price, etc.) (18, 19). Accordingly, based on the above views, the following hypothesis has been settled:

H2: *There is a strong correlation between food access and food availability*.

In its most basic form, food availability refers to the state in which food is produced to be consumed at local levels, where local people or households can easily find the food they need. It illustrates how different types of food are produced and supplied. Furthermore, the process of food availability is taken into account, mainly focusing on the dietary preferences of the consumer. These important variables are convenience, cost, taste, and cultural norms. In addition to these, there exist additional variables such as socioeconomic status and food accessibility, which essentially impact food purchases and nutrient quality (20).

According to other authors, food availability, food accessibility, and food consumption are critical to achieving food security (21).

Consumer decisions have a direct impact on nutrition and sustainability results, both of which are impacted by the type and amount of money spent and the diversity of food available (22). Also, consumers are developing more and more different behaviors as a direct consequence of technology impact and social media communications (23).

Since the availability of food is dependent upon both naturally occurring and sustainably farmed land systems, it is generally accepted that long-term food security requires an ecosystem-aware food security policy. Governments that practice sustainable land use and prudent resource management can support long-term, productive agriculture. In the same time, farmers should become more aware and adopt a green based design for the production process, along with green marketing techniques capable to generate a favorable mentality among consumers, highlighting their health benefits based on sustainable farming products consumption (24).

Allocating land tenure rights and access to natural resources, preserving soil and pollinators that are essential to crop growth, preserving forests that provide food sources and aid in water regulation, and permitting ecosystem restoration services to maintain healthy ecosystems are some of the specific policies that will help achieve these goals (25).

The food availability reduction can cause a reduction of food per capita supply, which is usually caused by natural disasters, wars or pandemics. The insufficient production and availability of food represent the main causes of famines and starvation (26).

At the same time, the availability of food is so important as it influences people by adjusting the pace at which they consume calories. An individual needs to sustain a positive or at least equilibrium energy balance over an extended period to stay healthy, even though their energy intake rates will constantly fluctuate across different time scales (27).

Moreover, the rising prevalence of food insecurity, in the last decade, has become a growing concern for many low- and middle-income countries (28). This issue has been exacerbated by natural disasters and socioeconomic instability (29), various factors are contributing to this alarming trend (e.g., conflict, global health matters, inflation), (30, 31). According to FAO (32), a situation where people lack adequate access (e.g., physical, social or economic) to nutritious food is referred to as food insecurity and it occurs when individuals do not have the necessary resources to meet their daily needs. Hence, based on the above understandings, the following three hypotheses have been determined:

H3: *Inadequate food consumption positively affects food insecurity*.
H4: *Inadequate food access positively affects food insecurity*.
H5: *Inadequate food positively affects food insecurity*.

Strategies should strive to reduce the environmental effect of the agricultural sector and adapt farming systems to the impacts of extreme climate change to manage the availability dilemma and achieve food security. High-yielding crop varieties, sustainable soil management techniques, the use of irrigation technology that improves water usage efficiency (like drip irrigation), and farmer training can all help farming systems adapt to climate change (17). Using integrated farming techniques could result in less reliance on outside inputs, which would benefit the environment (33).

In this sense, agroecological intensification can be quite beneficial. Agroecological intensification, for instance, can entail replacing chemical fertilizers with legumes and pesticides with biological pest control, such as employing predators (34). Precision farming techniques can lower waste and pollution in the environment in industrialized nations (35). These methods can lower the detrimental environmental externalities of agricultural farming systems, boost yields over time, and save significant production costs. Low-income nations lack sophisticated farm input markets, therefore utilizing biological processes and relying as little as possible on outside inputs could increase local productivity and guarantee food availability (33). As a trend, bio-economy has a strong pace due to the importance of the outcomes that are dealing with the preservation of bio-resources and the possibility to have a high degree of efficiency for environment related activities like farming (36).

“The access of all people, on a permanent basis, to the necessary food for an active and healthy life” is the definition of food security (37). Although there are several levels at which food security can be assessed, the majority of references are made to the global, national, and microeconomic—that is, to the family and individual—levels. One or more of the four components of food security—food availability, supply stability, economic access, and the individual's need for wholesome, nutrient-dense food—are highlighted, depending on the level of reference. Therefore, the ability of nations to offer an adequate agricultural supply to meet the population's food and nutritional demands is the main focus when applying the idea of food security at the global or national level (38). Simultaneously, newer strategies (39) emphasize “food autonomy” as a component of stable food security, which lessens susceptibility to changes in both domestic and international agricultural markets.

#### 2.1.1 Reviews related to food insecurity, food availability and food access among Romanian rural consumers

Food availability in the countryside is a crucial concern, not only in Romania but also in the whole world. The rural environment plays a critical role in food production, but there are still significant challenges in ensuring access to adequate food for rural communities.

In the European Union, Romania has the most subsistence farms per capita. Practically speaking, 3.3 million of Romania's 3.7 million farms can be classified as subsistence farms due to the incredibly low value of the produced goods. Despite playing a smaller part in the marketplace, these small farms are crucial to the rural community because they provide food and social security while also helping to preserve the environment by using conventional production techniques (40).

Romania has a varied pattern of food consumption because of the large proportion of its population living in rural areas. Thus, there are two patterns of food consumption: one for the urban population, where access to food is primarily determined by the purchasing power of the households, and another for the rural population, which consists of land-owning families whose purchasing power is determined by the ratio of the prices of goods sold on the farm to the prices of goods purchased on the market. It is clear that these consumption patterns are not pure forms because even the urban population exhibits high levels of self-consumption that are either directly or indirectly related to the household members' farming activities.

However, the availability of food does not ensure that it will be accessible, as issues with economic distribution in society can have a significant negative influence on both food security and access to food at the home level. Food security is therefore seen as a family or individual issue in the last instance. Generally speaking, hunger and food insecurity are a direct effect of poverty. Poor households will be able to afford and probably want to eat a sufficient diet as a result of economic growth and income increases (41). At the same time, periods of food deprivation (like historical well-known events – Dutch Hunger Winter, etc) can have undesired effects in a long run on the future generations' capacity to manage a healthy diet (42).

Access to food for rural households in Romania is contingent not just on household incomes but also on the agricultural resources these households possess initially. This is because the majority of Romanian peasant farms are small-scale households, that have inadequate market connections, and mostly use their produce for self-consumption.

Based on current estimates, 82% of Romanian farms produce mostly for their own consumption (43), whereas just 16.5% produce primarily for direct sales (44). In this way, subsistence agriculture, which makes up for the lack of monetary income and provides a nutritional standard for survival, appears to be a safety net for the impoverished population residing in rural areas as well as for certain urban households that own agricultural land. This occurs under the circumstances that our nation's families continue to consume food at relatively high percentages of their consumption expenditures, demonstrating how vulnerable all households are to agriculture—more specifically, to the prices of agricultural products on both the local and international markets.

Factors influencing the availability of food in rural areas, according to Vávra et al. (45), are as follows:

1. Agricultural infrastructure—The availability of food in rural areas is closely related to agricultural infrastructure. Access to resources such as quality seeds, and modern and appropriate agricultural machinery can significantly increase the production of food technologies. Climate change: Climate change can negatively affect agricultural production, with significant consequences for food availability. Extreme events such as drought or flooding can disrupt food supply chains.
2. Access to markets—For rural producers to sell their products, it is essential to have efficient transport systems and road infrastructure to facilitate access to markets.
3. Agricultural education—An agriculturally educated rural community can benefit from better farming and resource management techniques, thereby contributing to increased food production.
4. Agricultural policies—Government policies on agriculture can have a significant impact on food availability. Subsidies, financial support and regulations can influence how rural farmers operate and grow their businesses.

The specific context of Romania, according to the National Institute of Statistics is described by the following characteristics:

1. Traditional agricultural structure—Romania has a long agricultural tradition, and many rural communities continue to depend on agriculture for subsistence and income. However, there are some challenges related to the modernization of the agricultural sector.
2. Small producers—A large part of agricultural production in Romania comes from small agricultural producers. They may experience difficulties in accessing modern technologies and markets, thus affecting the availability of food.
3. Demographic changes—Population migration to cities can lead to aging rural communities and a decline in the agricultural workforce, which can affect food availability.
4. Rural development program—The Romanian government implements programs for rural development, with an emphasis on the modernization of agricultural infrastructure, the stimulation of ecological agriculture and the support of small producers.
5. Access to credits—Limited access to credits for farmers can be a barrier to the development of agricultural businesses and, implicitly, to ensuring an adequate availability of food in the countryside.

One of the most reliable sources of information about the factors influencing global food security is the Global Food Security Index (GFSI) (46). It assesses food security in 113 countries using four main criteria: price, availability, quality and safety, and sustainability and adaptation. It was created by Economist Impact with assistance from Corteva AgroSciences. A dynamic benchmarking model built from 68 qualitative and quantitative drivers of food security serves as the foundation for the index. Economist Impact chose the 113 countries in the index with consideration for regional variety, economic significance, population size (bigger countries were picked to reflect a larger proportion of the world's population), and the intention of incorporating all regions of the world.

According to GFSI, in 2022, in the overall ranking table, Romania was in the 45th position with a score of 68.8 out of 100, after all the EU countries. The first position in the ranking is held by Finland with a score of 83.7 points. While comparing its' position with the year 2012, Romania maintained the 45th position but improved the score with 5.8 points (47).

Still, the food consumption patterns of Romanians still reflect poverty, but the effects of poverty on population nutrition are more pronounced in urban areas than in rural ones.

More stable food availability results from the contact between agriculture and rural homes, despite the inferior quality features of the food (higher levels of alcohol, less animal protein, and a higher prevalence of fats of animal origin and high cholesterol).

Simultaneously, a reduced intake of meat, fresh vegetables, and fruit indicates an even less diverse food consumption in rural areas when we compare consumption by residential areas. The rural area's food intake is seasonal, with virtually little fruit and vegetable eating occurring outside of season (40).

The rising prevalence of food insecurity, in the last decade, has become a growing concern for many low- and middle-income countries (28). This issue has been exacerbated by natural disasters and socioeconomic instability (29), various factors are contributing to this alarming trend (e.g., conflict, global health matters, inflation), (30, 31). According to FAO (32), a situation where people lack adequate access (e.g., physical, social or economic) to nutritious food is referred to as food insecurity and it occurs when individuals do not have the necessary resources to meet their daily needs.

Even though farmers (i.e., small-scale farmers) are primarily responsible for ensuring food security in the national framework, they are still susceptible to the risk of food insecurity at home (48).

Previous researchers have considered the emergence of COVID-19 can worsen the diet quality and increase the intake of various food products. This could lead to future health problems and the promotion of nutritional awareness is needed (49, 50). Scholars from Romania noted the behavior of consumers prompted consumers to pay more attention to where their food comes from and also shifted their focus to buying local products and issues related to food waste (8, 40, 51–54).

Similarly, other Romanian authors, pointed out that for people residing in rural areas/small farms, the manifest attributes of food choice are classified into several constructs: price, quality, sustainable food, the impact of products on their health, the quantity of thrown food, accessibility of organic food, or Romanian traditional food (55–61).

Therefore, from the aspects previously described, it is noted that Romanian researchers treated less the effects of anxiety regarding the quality and quantity of food, caused by access to food of rural consumers in Romania.

Previous research from different sources has pointed out that easier access to supermarkets, measured in different settings, was associated with food consumption, particularly improved fruit or vegetable intakes or overall diet quality (12).

Likewise, previous research has indicated the relationship between food consumption and food availability at the local and national levels (62, 63). Thus, based on the above judgements, the following hypothesis has been established:

H6: *There is a strong correlation between food consumption and food availability*.

**Figure 1:**
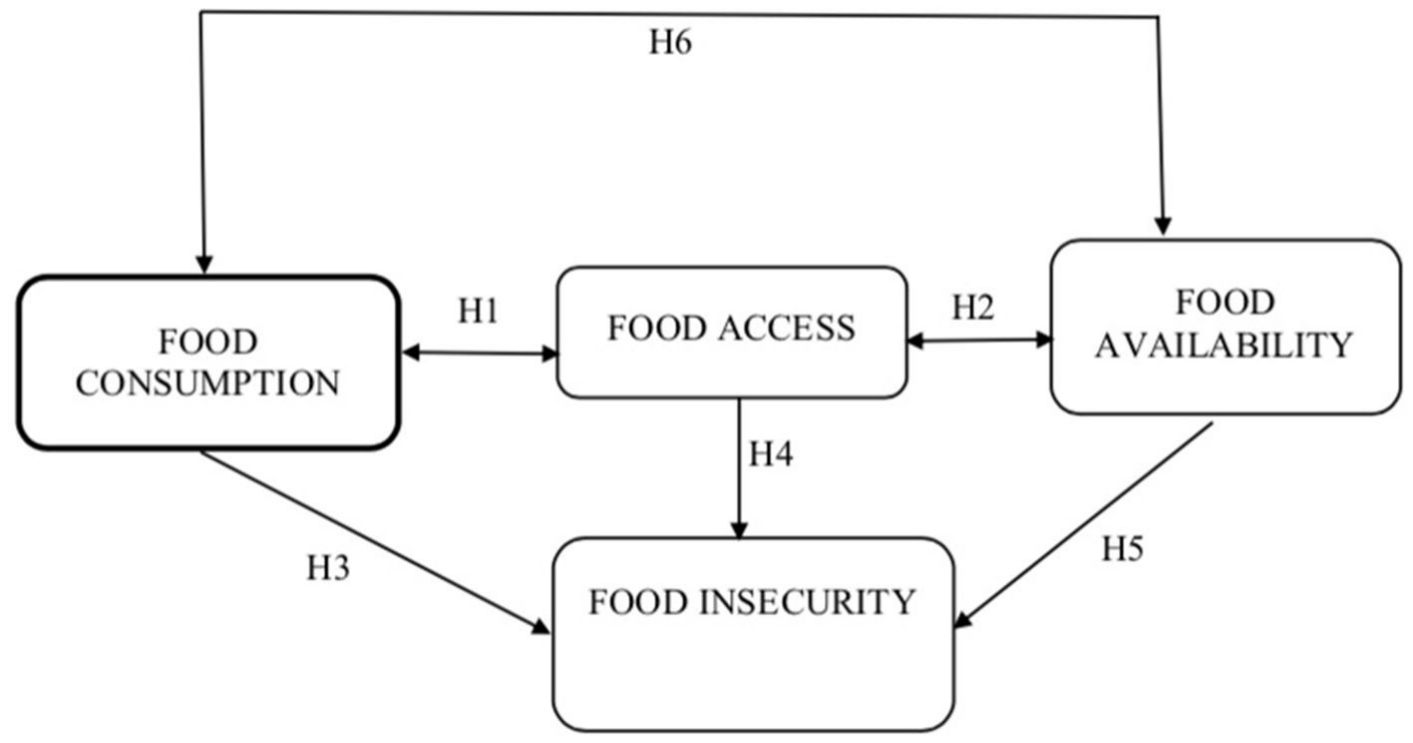
The proposed conceptual model.

After analyzing the literature, we considered the appropriate relationships among food consumption, food access, food availability and food insecurity in the form of the model proposed in Figure 1 (proposed conceptual model).

According to our conceptual model, we can outline that the negative balance of any of the three indicators (i.e., food consumption, food access, and food availability) leads to food insecurity. Therefore, the concept of food insecurity is based on three fundamental elements: (1) Inadequate food consumption, (2) Inadequate food availability; (3) Inadequate access to food.

### 2.2 Research methodology

The data collection was carried out by using the survey based on a questionnaire on a sample of 875 inhabitants from rural areas in Romania. The scales used in measuring the variables of this study were adapted from previous research and adjusted to fit the specific context of our study.

The surveys were conducted in Romania during 2021–2022. The sample included 900 people from rural areas from Transylvania, Moldova, and Dobrogea regions in Romania. The selection of the sample was random. In the selection of farms, the representativeness of the sample was followed. This representation was achieved by dividing the population evenly by region/sub-regions. Research data were collected in the form of direct interviews by interview operators.

The interview based on the questionnaire administered by the operators was a complex one including economic, social, sustainability, market links and job satisfaction aspects.

Regarding ethical approval, we highlight the fact that the questionnaire text mentioned the guarantee that technical and procedural measures have been taken to protect and ensure the confidentiality, integrity and accessibility of processed personal data and also that unauthorized use or access and personal data breach will be prevented, in accordance with the legislation in force.

Incomplete and invalid questionnaires were removed from the sample and 875 valid questionnaires were obtained.

According to USAID Title II and Child Survival and Health Grant indicators of the access component of household food insecurity [hereafter referred to as household food insecurity (access)] can be used to guide, monitor and evaluate in distinction countries. Over the past several years, USAID's Food and Nutrition Technical Assistance (FANTA) project has supported a series of research initiatives to explore and test different options for meeting this need (64).

The Household Food Insecurity Access Scale (HFIAS), is an adaptation of the approach used to estimate the prevalence of food insecurity in the United States (U.S.) annually. The method is based on the idea that the experience of food insecurity (access) causes predictable reactions and responses that can be captured and quantified through a survey and summarized in a scale. Version 3 of the guide, the HFIAS questions have been refined to address the recommendations of the Nutrition and Consumer Protection Division, Food and Agriculture Organization of the United Nations (FAO) (64).

U.S. Household Food Security Survey Module (US HFSSM) asks respondents to describe behaviors and attitudes that relate to these various aspects of the food insecurity experience (65). A question relating to perceptions of insufficient *quantity* asks whether any adults had to eat less than they thought they should. The US HFSSM are summarized in a scale to provide a continuous indicator of the degree of a household's food insecurity.

FANTA and its partners they defined a set of questions (Household Food Insecurity Access Scale Generic Questions) that have been used in several countries and appear to distinguish the food secure from the insecure households across different cultural contexts (64).

The categorical variables are used in separate questions to describe the structure of the sample and to explain the correlations between the independent variables and the dependent ones. The responses were assessed on a 3-point Likert-type scale ranging from 1 = rarely (once or twice in the past four weeks) 2 = sometimes (three to ten times in the past four weeks) 3 = often (more than ten times in the past four weeks) in accord with HFIAS scale (64). The measured variables were food consumption, food access, food availability and food insecurity. The proposed model is based on the HFIAS scale and the proposed items to measure food availability and food consumption by Pandey and Bardsley (66).

In our research, food access, was measured using: (i) Anxiety about the household food supply (FAA); (ii) Perceptions that food is of insufficient quality, which includes food variety, and preferences (FAV); (iii) Perception that food is of insufficient food quantity, which includes food supply (FAQ).

For analyzing food consumption were used: (i) Income (FCI); (ii) Production of food (FCPF) and (iii) health, social safety nets (FCS) (66).

Food availability was measured with the variables: (i) Resources for food—natural, human, and physical, (ARF) and (ii) availability Production of food—production, food imports, market integration (APF).

The HFIAS questions relate to three different domains of food insecurity (access) found to be common to the cultures examined in a cross-country literature review (67, 68).

The questionnaire includes 15 questions with specific content for the three constructs of the model (Table 1).

**Table 1:** Variable measures.

**Model construct**	**Exogenous variables**	**Items**	**Sources**
Food access	Anxiety and uncertainty about the household food supply: (FAA)	Did you worry that your household would not have enough food?	(64)
	Insufficient quality, which includes food variety, and preferences (FAV)	Did you or any household member have to eat some foods that you really did not want to eat because of a lack of resources to obtain other types of food?	
	Insufficient food quantity (FAQ)	Did you or any household member have to eat fewer meals in a day because there was not enough food?	
Food consumption	Income (FCI)	Level of income	(66)
	Production of food (FCPF)	Production, purchasing power, social	
	Health, social safety nets (FCS)	Safety nets, community support	
Food availability	Resources for food (ARF)	Natural, human, and physical	(66, 69)
	Availability Production of food (APF)	Food production, market integration	

The structure of the sample is described using data represented in Table 2.

**Table 2:** Sample structure.

**Variable**	**Category**	**Frequency**	**Percentage**
Gender	Male	633	72.3%
	Female	242	27.7%
Agricultural Education	Yes	484	55.3%
	No	391	44.7%
Age	18–35	138	15.77%
	36–45	327	37.37%
	46–55	189	21.60%
	56–65	137	15.66%
	Over 66	84	9.6%
Number of household members	2–4	548	62.63%
	4–8	284	32.46%
	Over 8	43	4.91%

Therefore, a model was created with the following dimensions: food consumption, food access, food availability, and food insecurity.

The components of the model were analyzed using the exploratory factor analysis method. This method ensures accuracy and contributes to defining the model as correctly as possible and identifying the component variables that could be removed from the analysis to reduce the information that must be analyzed without affecting the accuracy of the final result. The Cronbach's Alpha test is used to assess the reliability of the scales. For each factor, Cronbach's alpha coefficient was calculated to measure the internal consistency. It measures the sum of observed variables associated with the overall variable to eliminate low correlation coefficient observation variables to the overall variable. For exploratory analysis, it is essential to conduct a test of the scale's reliability using Cronbach's Alpha. Although it is not possible to discuss a specific value that Cronbach's alpha coefficient can have to guarantee a high degree of fidelity of the measurements, several researchers suggest that values that are ≥0.90 can be considered excellent, while values ≥0.80 may be considered good and those ≥0.70 are acceptable (70, 71).

## 3 Results

According to the results, Cronbach's alpha coefficient values are above 0.90, for each component measurement (a value of 0.993 for food utilization, a value of 0.998 for food access, a value of 0.995 for food availability 0.995) which means that the fidelity (consistency) of the scales in case of latent variables is confirmed (71, 72).

In the case of The Kaiser–Meyer–Olkin (KMO) test for measuring the suitability of the sample, it must have a minimum workload of 0.5 to consider that the sample size is appropriate for performing the factor analysis and over 0.7 data adequacy is considered very good. According to the results Kaiser–Meyer–Olkin (KMO) for food consumption is 0.776, food access 0.771 and food availability 0.5 for the latter, the suitability is moderate (73).

Another condition to be able to apply exploratory type factorial analysis and the main components analysis procedure deals with homoscedasticity verification or homogeneity of variances by the Bartlett test. This test is sensitive to abnormalities. The Bartlett Test is used for the null hypothesis test that implies all population variations are equal, compared to the alternative hypothesis that assumes at least two are different. In other words, the Bartlett Test examines whether the correlation matrix of the investigated population is similar to the identity matrix. If the population correlation matrix resembles the identity matrix, then it means that each variable correlates poorly with all other variables. This test is considered significant and the null hypothesis is rejected if *p* < 0.001 (Table 3). Values regarding exploratory factor analysis (values of Bartlett's Test of Sphericity, Kaiser–Meyer–Olkin test and Cronbach's alpha coefficient, for each dimension of the model, extracted and retained based on the considered items).

**Table 3:** Values regarding exploratory factor analysis.

**Test statistic**	**Food consumption**	**Food access**	**Food availability**	**Food insecurity**
Cronbach's Alpha	0.993	0.998	0.995	0.973
Bartlett's test of sphericity^*^	6,128.625	8,175.134	3,382.031	7.350
Approx. Chi-Square df Sig.	3.000	3.000	1.000	1.000
Kaiser–Meyer–Olkin Measure of Sampling Adequacy	0.776	0.771	0.500	0.760

^*^Extraction method Principal component analysis.

In conclusion, it can be stated that factor analysis can be used because the latent variables determined to start from the initial items are valid in terms of item commonality (Kaiser–Meyer–Olkin test), item sphericity (Bartlett Test) and measurement scale consistency (Cronbach's alpha). A confirmatory factor analysis was conducted using version 28.0 of the IBM-SPSS AMOS program.

Table 4 presents the goodness of fit and we point out that indices of the structural model were satisfactory for the variables of food consumption, food access, food availability and food insecurity (Chi-square–CMIN = 59.659, df = 22; p = 0.00; GFI = 0.934; IFI = 0.996, NFI = 0.995, TLI = 0.993, CFI = 0.996, RMSEA = 0.063).

**Table 4:** Fit indices for the model.

**Model**	***P***	**GFI**	**AGFI**	**NFI**	**RFI**	**IFI**
Research obtained values	0.000	0.934	0.912	0.995	0.992	0.996
Theoretical statistical values^*^	< 0.05	>0.90	>0.90	>0.95	>0.90	>0.90
**Model**	**TLI**	**CFI**	**PNFI**	**PCFI**	**RMSEA**	**PCLOSE**
Research obtained values	0.993	0.996	0.608	0.609	0.063	0.00
Theoretical statistical values	>0.95	>0.95	>0.50	>0.50	< 0.1	< 0.05

^*^Statistical theoretical values are considered according to Hooper et al. (74).

Comparing the values obtained in Table 4 with the limit values of each index, it can be stated that the proposed model is satisfactory in terms of statistical consistency (Table 5).

**Table 5:** Standardized direct effect coefficient.

**Hypotheses**	**Correlations**	**β**	***P***	**Std. error**	**C.R**.	**Decision**
H1	FC → FACC	0.91	0.000	0.044	20.811	Supported^*^
H2	FACC → FA	0.91	0.000	0.044	20.786	Supported^*^
H3	FC → FIN	0.33	0.000	0.038	8.684	Supported^*^
H4	FACC → FIN	0.40	0.000	0.034	11.764	Supported^*^
H5	FA → FIN	1.07	0.000	0.276	3.870	Supported^*^
H6	FC → FA	0.92	0.000	0.047	20.878	Supported^*^

^*^Significant at CR > 1.96, p < 0.01.

One can notice from Table 4 that *p* < 0.01; the statistical significance of the parameter estimates test of the critical ratio (C.R.) needs to be >1.96 (75, 76, p. 494–505).

The general analysis of the model results shows that food consumption, food accessibility and food availability it influences directly food insecurity.

Hypotheses H1 - Food consumption has a direct positive and significant effect on food access β = 0.91, *p* < 0.01, Critical Ratio test = 20.811 > 1.96 is accepted. Income level, purchasing power, safety nets, and community support had a direct and significant effect on Anxiety and uncertainty about the household food supply, insufficient quality, which includes food variety, and preferences and insufficient food quantity.

Also, there is a strong correlation between food access and food availability (β = 0.91, *p* < 0.01, CR test = 20.786 > 1.96), and the H2 hypothesis is supported. Food availability has a direct positive and significant effect on food access. Resources for the food and the availability and production of food have a direct influence on food acces.

Hypothesis H3 Inadequate food consumption has a direct positive and significant effect on food insecurity, β = 0.33, *p* < 0.01, and Critical Ratio test = 8.684. The correlation is not very strong, but according to the data, the hypothesis is supported.

Hypothesis H4 Inadequate food access has a direct positive and significant effect on food insecurity perception, β = 0.40, *p* < 0.01, and CR =11.764. Even though the correlation is not very strong, H3 is supported.

Hypothesis H5 Inadequate food availability has a direct positive and significant effect on food insecurity perception β = 1.07, *p* < 0.01, and Critical Ratio test =3.870. Similarly, the correlation is very strong and H5 is supported.

For hypothesis H6 we noticed that Adequate food availability has a direct positive and significant effect on food consumption β = 0.92, p < 0.01, and CR =20.872.

## 4 Discussion

The different types of households, complex social structures and the inequitable distribution of resources among its members reinforce the idea that the concept of “household food security” has political relevance. The research carried out to date has not yet provided an adequate perspective on individual food insecurity. The measurement of different experiences of insecurity within the household should be continued and the implications of these results discussed.

One of the greatest challenges for specialists has been to develop a set of criteria that can be used to assess the validity of adapted scales of experiential food insecurity in different cultures without complete data sets.

This research has identified highly variable food insecurity situations in Romania.

According to USAID, three distinct variables are essential to the attainment of food insecurity: (1) Food Availability: if there are insufficient quantities of appropriate, necessary types of food from domestic production; (2) Food Access: individuals have inadequate incomes or other resources to purchase or barter to obtain levels of appropriate food needed to maintain consumption of an adequate diet/nutrition level; and (3) Food Consumption: food is improperly used, improper food processing and storage techniques are employed, inadequate knowledge of nutrition and child care techniques exist and is applied, and adequate health and sanitation services.

Having as a model the household food insecurity access scale (i.e., HFIAS) used in the USA to measure food insecurity, we created a model that can be used at the level of rural households in Romania.

The investigated population were small farms, households that deal with agriculture and that produce their basic food. In the case of the small farmer, the indicators from the HFIAS model were adapted. For small households, food diversification is a problem for the population and limited access to other foods than those in the household.

Dimensions of food insecurity were measured with items from the scale HFIAS (67, 68) combined with the model proposed by Pandey and Bardsley (66) and FAO and SAARC (69). With the HFIAS scale, we measured the food access and food availability and food consumption the proposed models were adapted.

Likewise, we discover that the perception of *uncertainty* or *anxiety* over food is less in the case of the investigated population from Romania compared to the studies done in the USA, or other countries (77).

The food consumption decisions are influenced in a great measure by different factors among them being the perception of food products labels that can elicit a positive impact in the long run in case of subsequent loyal behavior (78, 79).

The specialized literature analyzed refers to the use of food insecurity measurement scales in a comparative way in different time intervals. The research carried out at the level of consumers in rural areas in Romania requires a reiteration of the study to be able to analyse and compare the results.

## 5 Conclusions

In agreement with USAID studies and partially using the HFIAS scale, we consider that food insecurity is perceived by the inhabitants of Romanian rural households as being strongly negatively influenced by food availability, but less influenced by food consumption and access.

The rural population has direct access to basic foods, to food subject to production and valorisation in their small farms. Inadequate access to food is due to infrastructure. The level of anxiety about the quality or quantity of food is closely related to the geographical area, being influenced by crops and access to imported products.

Food must be available for households to have access to, and a household must have access to food for individual household members to have appropriate food utilization/consumption. All three elements of food security must be achieved for food security to be attained (77).

That situation has increased local dependencies on food supplied from distant locations, with households having little control over prices received for their produce or paid for food, whereas risks increase as the diversity of their food production and consumption systems decline (80). Changing environmental and socio-economic conditions could worsen that situation (66, 81).

## Data availability statement

The datasets presented in this article are not readily available because confidentiality restrictions. Requests to access the datasets should be directed to bologandreea@gmail.com.

## Author contributions

CP: Formal analysis, Methodology, Writing – original draft, Writing – review & editing. SM: Investigation, Writing – review & editing. ID: Investigation, Methodology, Writing – original draft, Writing – review & editing. AM: Conceptualization, Investigation, Writing – original draft. CH: Writing – original draft, Writing – review & editing.
